# Optimizing the plasma oxidation of aluminum gate electrodes for ultrathin gate oxides in organic transistors

**DOI:** 10.1038/s41598-021-85517-7

**Published:** 2021-03-18

**Authors:** Michael Geiger, Marion Hagel, Thomas Reindl, Jürgen Weis, R. Thomas Weitz, Helena Solodenko, Guido Schmitz, Ute Zschieschang, Hagen Klauk, Rachana Acharya

**Affiliations:** 1 Max Planck Institute for Solid State Research, Stuttgart, Germany; 2 The 1st Physical Institute, University of Göttingen, Göttingen, Germany; 3 Faculty of Physics, Ludwig-Maximilians-University, München, Germany; 4 Institute of Materials Science, University of Stuttgart, Stuttgart, Germany

**Keywords:** Surfaces, interfaces and thin films, Electronic properties and materials, Electronic devices, Semiconductors

## Abstract

A critical requirement for the application of organic thin-film transistors (TFTs) in mobile or wearable applications is low-voltage operation, which can be achieved by employing ultrathin, high-capacitance gate dielectrics. One option is a hybrid dielectric composed of a thin film of aluminum oxide and a molecular self-assembled monolayer in which the aluminum oxide is formed by exposure of the surface of the aluminum gate electrode to a radio-frequency-generated oxygen plasma. This work investigates how the properties of such dielectrics are affected by the plasma power and the duration of the plasma exposure. For various combinations of plasma power and duration, the thickness and the capacitance of the dielectrics, the leakage-current density through the dielectrics, and the current–voltage characteristics of organic TFTs in which these dielectrics serve as the gate insulator have been evaluated. The influence of the plasma parameters on the surface properties of the dielectrics, the thin-film morphology of the vacuum-deposited organic-semiconductor films, and the resulting TFT characteristics has also been investigated.

## Introduction

Organic thin-film transistors (TFTs) are being developed for flexible electronics applications, such as rollable or foldable active-matrix displays and conformable sensors^1–5^. To ensure the safe handling and portable nature of these systems, they will typically be powered by small batteries or solar cells and will thus be operating at low voltages of about 2 to 3 V. To enable organic TFTs to operate with low voltages, the gate dielectric should have a large unit-area capacitance. Examples of gate dielectrics suitable for low-voltage organic TFTs include thin insulating polymers^6,7^, high-permittivity insulating metal oxides^8,9^, self-assembled nanodielectrics^10,11^, and ultrathin hybrid dielectrics composed of a thin metal oxide in combination with a molecular self-assembled monolayer (SAM)^12,13^. The thickness of these dielectrics must be sufficiently small to provide a large unit-area capacitance and thereby low-voltage TFT operation^14–17^, but sufficiently large to suppress undesirable charge leakage and thus allow for low-power circuit and system operation^18–20^. In addition, the dielectrics should be sufficiently robust to allow the TFTs to be fabricated on unconventional and potentially rough substrates, such as plastics and paper^21–23^.

This work focuses on ultrathin hybrid gate dielectrics. The first component of these dielectrics is a thin metal oxide that can be produced by atomic layer deposition^8,24,25^, anodic oxidation^26–28^, UV/ozone-assisted oxidation^29–31^, or plasma-assisted oxidation of the surface of the gate electrode^13^. Among the advantages of the plasma-oxidation process are the fact that it does not require electrical contact to the gate metal during the oxidation process^32^ (which greatly simplifies the fabrication process), that the oxide is formed only where needed for the TFTs (which eliminates the need for subtractive patterning to open vias for interconnects^22^) and that the high quality of the native interface between the gate metal and the gate oxide minimizes the hysteresis in the current–voltage characteristics and the subthreshold swing of the TFTs^33,34^. The most popular material combinations for the gate metal and the gate oxide are aluminum/aluminum oxide^15,35^ and titanium/titanium oxide^14,27^.

The second component of these hybrid dielectrics is a SAM of organic molecules that are composed of an anchor group to facilitate chemisorption on the metal-oxide surface and an aliphatic tail to facilitate the self-assembly of a well-ordered molecular monolayer^36^. The preferred anchor group for chemisorption on aluminum oxide is the phosphonic acid^37^, while the aliphatic tail can be an alkyl or fluoroalkyl chain^25,38–41^. The effects of the properties of the SAM-forming molecules, such as the alkyl or fluoroalkyl chain length^31,42–44^, and the details of the SAM-formation process have been investigated in great detail in the past^45–48^. One result of these studies is that the best TFT performance is often obtained with a medium-chain-length alkylphosphonic acid, such as *n*-tetradecylphosphonic acid, processed from solution.

In this work, we focused on the metal-oxide component of the hybrid dielectric. We prepared thin films of aluminum oxide (AlO_x_) by exposing the surface of vacuum-deposited aluminum films to a capacitively coupled radio-frequency (13.56 MHz) plasma in pure, low-pressure oxygen and investigated the extent to which the properties of the resulting AlO_x_ films can be tuned by adjusting two of the parameters of the plasma-oxidation process, namely the plasma power and the duration of the plasma exposure. We varied the plasma power from 10 to 300 W and the plasma duration from 10 to 1800 s and studied how this affects the properties of the AlO_x_ films and those of organic TFTs in which these AlO_x_ films serve either as the gate dielectric or as the first component of a hybrid AlO_x_/SAM gate dielectric. The thickness of the plasma-grown AlO_x_ films was determined by transmission electron microscopy (TEM). For both the bare-AlO_x_ and the hybrid AlO_x_/SAM dielectrics, we measured the unit-area capacitance, the leakage-current density, the surface properties and the current–voltage characteristics of organic TFTs fabricated in the inverted staggered (bottom-gate, top-contact) architecture using the vacuum-deposited small-molecule semiconductor dinaphtho[2,3-b:2′,3′-f]thieno[3,2-b]thiophene (DNTT)^49–51^ and analyzed the effects of the plasma parameters on these material and device characteristics.

## Results and discussion

### Thickness of plasma-grown AlO_x_ films

**Figure 1:**
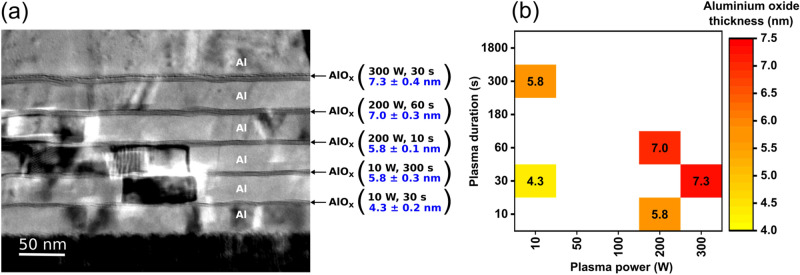
Thickness of plasma-grown aluminum oxide films. (**a**) Cross-sectional TEM image indicating the thicknesses of five AlO_x_ films produced sequentially by the plasma-assisted surface oxidation of aluminum using five different combinations of plasma power and plasma duration. (**b**) Summary of the results.

Figure 1a shows a cross-sectional TEM image of a specimen prepared on a silicon substrate by repeating the deposition of 30-nm-thick aluminum and the plasma-assisted oxidation of the aluminum surface five times, each time with a different combination of plasma power and plasma duration. During each transfer of the substrate from the metal-deposition system to the plasma system, the aluminum surface was necessarily exposed to ambient air, causing the spontaneous formation of a native oxide film with a thickness of approximately 3 nm on the aluminum surface^52^. The TEM image indicates that after the plasma-assisted oxidation, the aluminum oxide films have a thickness of approximately 4.3 to 7.3 nm, depending on the plasma parameters. (The method of extracting the thickness of the AlO_x_ films from the TEM image is detailed in Fig. S1).

The lower limit of this thickness range (4.3 nm) is thus larger by approximately 1.3 nm than the thickness of the native oxide. Although this increase in the oxide thickness produced by the plasma process is quite small, it is of critical importance for the proper operation of organic TFTs in which these dielectrics are used as the gate insulator. Figure S2 shows the measured transfer characteristics of DNTT TFTs fabricated using bare-AlO_x_ and hybrid AlO_x_/SAM gate dielectrics based on native aluminum oxide (obtained without plasma process), and as can be seen, these TFTs either do not show a field effect (bare AlO_x_) or suffer from prohibitively large gate currents (hybrid AlO_x_/SAM dielectric).

The relation between the plasma parameters and the thickness of the plasma-grown AlO_x_ films extracted from the TEM image is illustrated in Fig. 1b. As expected, both a larger plasma power (by virtue of a higher kinetic energy of the oxygen radicals impinging on the oxide surface) and a longer plasma duration (by virtue of a larger number of incident radicals) result in thicker AlO_x_ films.

### Electrical properties of plasma-grown AlO_x_ dielectrics

**Figure 2:**
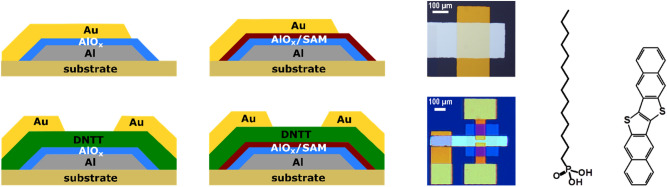
Schematic cross sections and photographs of metal–insulator–metal capacitors and bottom-gate, top-contact TFTs in which the insulator or gate dielectric is either a film of plasma-grown AlO_x_ or a combination of plasma-grown AlO_x_ and an *n*-tetradecylphosphonic acid SAM. Also shown are the chemical structure of *n*-tetradecylphosphonic acid and of the organic semiconductor dinaphtho[2,3-b:2′,3′-f]thieno[3,2-b]thiophene (DNTT).

To investigate how the electrical properties of plasma-grown AlO_x_ dielectrics and of hybrid AlO_x_/SAM dielectrics are affected by the plasma parameters, we fabricated metal–insulator–metal capacitors and bottom-gate, top-contact DNTT TFTs with dielectrics prepared using fifteen different combinations of plasma power (ranging from 10 to 300 W) and plasma duration (ranging from 10 to 1800 s). The devices were fabricated on silicon substrates coated with 100-nm-thick silicon dioxide. For the bottom electrode of the capacitors and the gate electrode of the TFTs, aluminum with a thickness of 30 nm and a root-mean-square surface roughness of less than 1 nm (measured by AFM^53^) was deposited by vacuum evaporation. AlO_x_ was produced by plasma oxidation, SAMs of *n*-tetradecylphosphonic acid were formed from solution, and DNTT was deposited by vacuum sublimation. For the top electrode of the capacitors and the source/drain contacts of the TFTs, gold was deposited by vacuum evaporation. Schematic cross sections and photographs of the capacitors and TFTs and the chemical structures of *n*-tetradecylphosphonic acid and DNTT are shown in Fig. 2.

**Figure 3:**
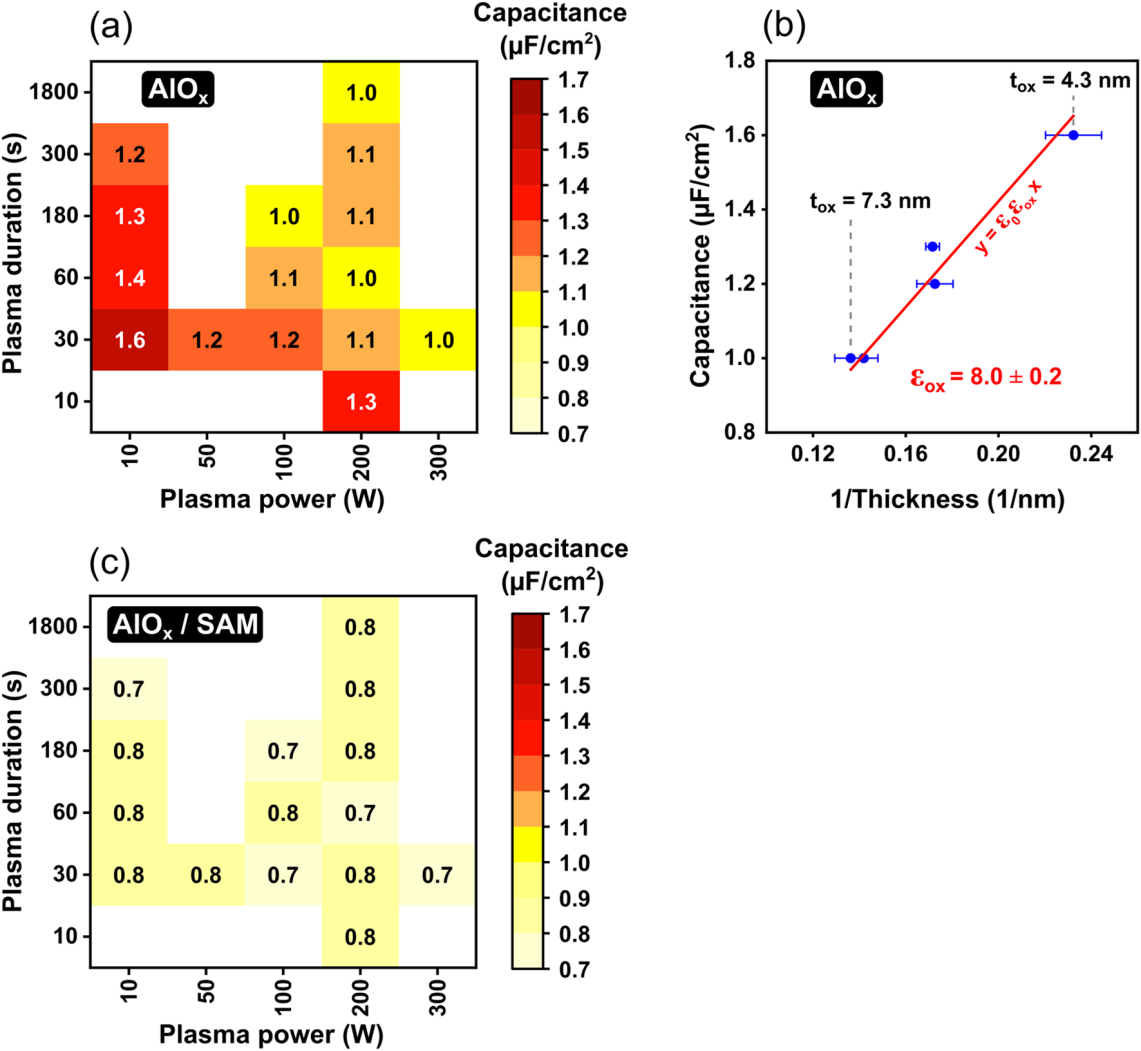
Capacitance of plasma-grown AlO_x_ and hybrid AlO_x_/SAM dielectrics. (**a**) Measured unit-area capacitance of capacitors with a bare-AlO_x_ dielectric as a function of plasma power and plasma duration. (**b**) Unit-area capacitance of bare-AlO_x_ dielectrics plotted as a function of the inverse of the AlO_x_ thickness determined by TEM (Fig. 1) to calculate the relative permittivity of the plasma-grown AlO_x_. The red line is a linear fit of Eq. (1) that was forced to pass through the origin (1/t_ox_ = 0; C_ox_ = 0). (**c**) Measured unit-area capacitance of capacitors with a hybrid AlO_x_/SAM dielectric as a function of plasma power and plasma duration.

The measured unit-area capacitance of the capacitors with a bare-AlO_x_ dielectric is plotted as a function of the plasma power and the plasma duration in Fig. 3a. Depending on these parameters, the unit-area capacitance varies from 1 to 1.6 µF/cm^2^, with the general trend of higher power and longer duration producing AlO_x_ films with smaller capacitance. In Fig. 3b, we plot the unit-area capacitance measured for each of the five plasma-parameter combinations for which the AlO_x_ thickness was determined by TEM (Fig. 1) as a function of the inverse of that thickness. The error bars reflect the accuracy of the method by which the oxide thickness was extracted from the TEM image (see Fig. S1). By fitting the measurement data with the theoretical relation between the unit-area capacitance C_ox_ and the oxide thickness t_ox_:

1 $${\mathrm{C}}_{\mathrm{ox}}= {\upvarepsilon }_{0}{\upvarepsilon }_{\mathrm{ox}} \frac{1}{{\mathrm{t}}_{\mathrm{ox}}}$$

(where ε_0_ is the vacuum permittivity and ε_ox_ the relative permittivity of the plasma-grown oxide) and forcing the linear fit through the origin (1/t_ox_ = 0; C_ox_ = 0), we obtain a value of 8 ± 0.2 for the relative permittivity of the plasma-grown AlO_x_ films. This result is in good agreement with the relative permittivity reported in the literature for aluminum oxide films produced by various methods^54–56^.

We note that a more elaborate analysis of the relation between the oxide thickness and the oxide capacitance would have included measurements of the oxide thickness for all fifteen combinations of plasma power and plasma duration shown in Fig. 3a, as opposed to selecting only five of these fifteen combinations for the TEM measurements. However, as this was not possible, we deliberately selected for the TEM analysis five plasma-parameter combinations covering both the extremes and the center of the range of thicknesses and capacitances as much as possible.

Closer inspection of Fig. 3a reveals that the influence of the plasma duration on the capacitance is relatively small, as long as the plasma power is at least 50 W and the duration is at least 30 s. For example, for a plasma power of 200 W, a unit-area capacitance of 1 µF/cm^2^ is obtained for a plasma duration of one minute and for a plasma duration of half an hour. Combined with the TEM results (Fig. 1), this suggests that the thickness of the plasma-grown AlO_x_ films saturates at a value of approximately 7 nm after a plasma duration of 30 to 60 s, provided the plasma power is at least 50 W. According to the Cabrera-Mott model^57^, this self-limiting oxide-growth behavior results from the low electronic conductivity of aluminum oxide and the small diffusivity of oxygen in aluminum oxide, which prevents oxygen from reaching the metal surface once the oxide thickness has reached a certain value determined mainly by the plasma power^57–59^.

Compared with the dependence of the capacitance on the plasma duration, its dependence on the plasma power appears to be more monotonic, but overall, the range over which the capacitance of the plasma-grown AlO_x_ can be tuned is nevertheless quite small (less than a factor of two). One benefit of this small range of accessible capacitances is that it renders the fabrication process more robust by suppressing the effects of unintended process-parameter variations on the resulting TFT characteristics.

**Figure 4:**
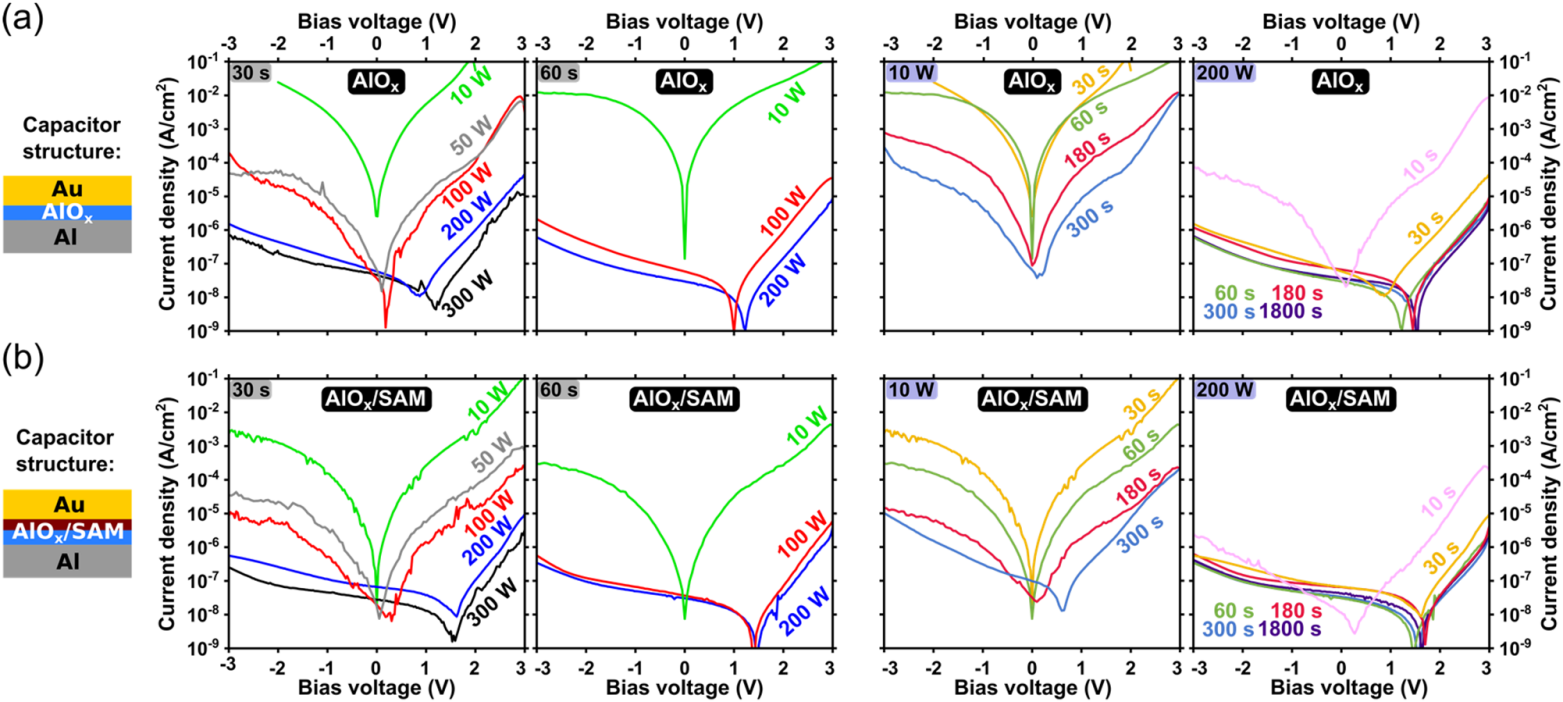
Current density measured as a function of applied voltage through capacitors with a bare-AlO_x_ dielectric (**a**) and a hybrid AlO_x_/SAM dielectric (**b**) for various combinations of plasma power and plasma duration.

In addition to the capacitance, we also measured the current–voltage characteristics of the capacitors to analyze the influence of the plasma power and duration on the leakage-current density through the dielectrics. The results for the capacitors with a bare-AlO_x_ dielectric are summarized in Fig. 4a. The general trend is similar to the one seen for the capacitance in Fig. 3a and is consistent with the TEM results: a higher plasma power and a longer plasma duration lead to thicker AlO_x_ films characterized by smaller leakage-current densities.

When the plasma power is smaller than about 50 to 100 W and the plasma duration is shorter than about 30 to 60 s, the leakage-current density through the bare-AlO_x_ films exceeds 10^–4^ A/cm^2^ at ± 3 V. On the other hand, for a plasma power of 200 W and a plasma duration of 60 s, the current density through the bare-AlO_x_ dielectrics is below 10^–5^ A/cm^2^ at ± 3 V. Figure 4a also shows that it is not possible to produce bare-AlO_x_ dielectrics by plasma oxidation that provide leakage-current densities significantly below 10^–6^ A/cm^2^ at ± 3 V.

### Electrical properties of hybrid AlO_x_/SAM dielectrics

Although it is possible to use the bare, plasma-grown AlO_x_ films discussed above as the gate dielectric for organic TFTs^40,60,61^, the often-preferred option is a hybrid gate dielectric in which the AlO_x_ is complemented by a phosphonic-acid SAM. This has a number of advantages, including a smaller density of interface trap states^62^, a smaller leakage-current density^40^, an improved bias-stress stability due to the expulsion of water from the semiconductor-dielectric interface^63^, the suppression of Fröhlich polarons due to the low permittivity of the organic SAM^64^, the possibility to tune the threshold voltage of the TFTs^15,25,38,65,66^, and the possibility to tune the surface energy of the dielectric.

In Fig. 3c, the unit-area capacitance of the capacitors with a hybrid AlO_x_/SAM dielectric is plotted as a function of the plasma power and plasma duration. Due to the additional contribution of the SAM, the capacitance of the hybrid AlO_x_/SAM dielectric (0.7–0.8 µF/cm^2^) is smaller than that of the bare-AlO_x_ dielectric (1–1.6 µF/cm^2^) and shows a notably smaller dependence on the plasma power and duration. From the measured unit-area capacitances of the bare-AlO_x_ and the hybrid AlO_x_/SAM dielectrics, the unit-area capacitance of the SAM can be estimated using the equation 1/C_total_ = 1/C_ox_ + 1/C_SAM_ (where C_total_, C_ox_ and C_SAM_ are the unit-area capacitances of the hybrid AlO_x_/SAM dielectric, the bare-AlO_x_ film, and the SAM, respectively), which yields values between approximately 1.7 and 2.1 µF/cm^2^ for the SAM, depending on the set of plasma parameters for which the calculation is performed. However, this does not mean that SAMs formed on AlO_x_ films produced with different plasma parameters have different capacitances, but merely reflects the uncertainty in the measured capacitances combined with the fact that the influence of variations in the SAM capacitance on the total capacitance is very small. Assuming that the molecules employed for the SAM (*n*-tetradecylphosphonic acid) have an alkyl-chain length of 1.9 nm, as reported previously^13,31^, and that the molecules in the SAM have a tilt angle of 20–30° with respect to the surface normal^13^, this corresponds to a permittivity between approximately 3.1 and 4.3 for the *n*-tetradecylphosphonic acid SAMs, which is larger by about 25 to 75% than the value of 2.5 that was reported previously for *n*-octadecyltrichlorosilane SAMs^67^. Whether this difference is systematic or not, and if so, how this difference can be explained, is not known.

The results of the measurements of the current density through the hybrid AlO_x_/SAM dielectric are summarized in Fig. 4b. As can be seen, the leakage-current density through the hybrid AlO_x_/SAM dielectrics is smaller by approximately an order of magnitude than the current density through the bare-AlO_x_ dielectrics, regardless of the plasma parameters. For a plasma power of 200 W and a duration of 60 s, the leakage-current density through the hybrid AlO_x_/SAM dielectric drops below 10^–6^ A/cm^2^. While this confirms the beneficial effect of the SAM in improving the insulating properties of the gate dielectric for low-power organic TFTs, the results in Fig. 4b also clearly demonstrate the critical importance of providing an optimized AlO_x_ film, even when complementing it with a SAM.

### Organic TFTs with bare-AlO_x_ and hybrid AlO_x_/SAM gate dielectrics

**Figure 5:**
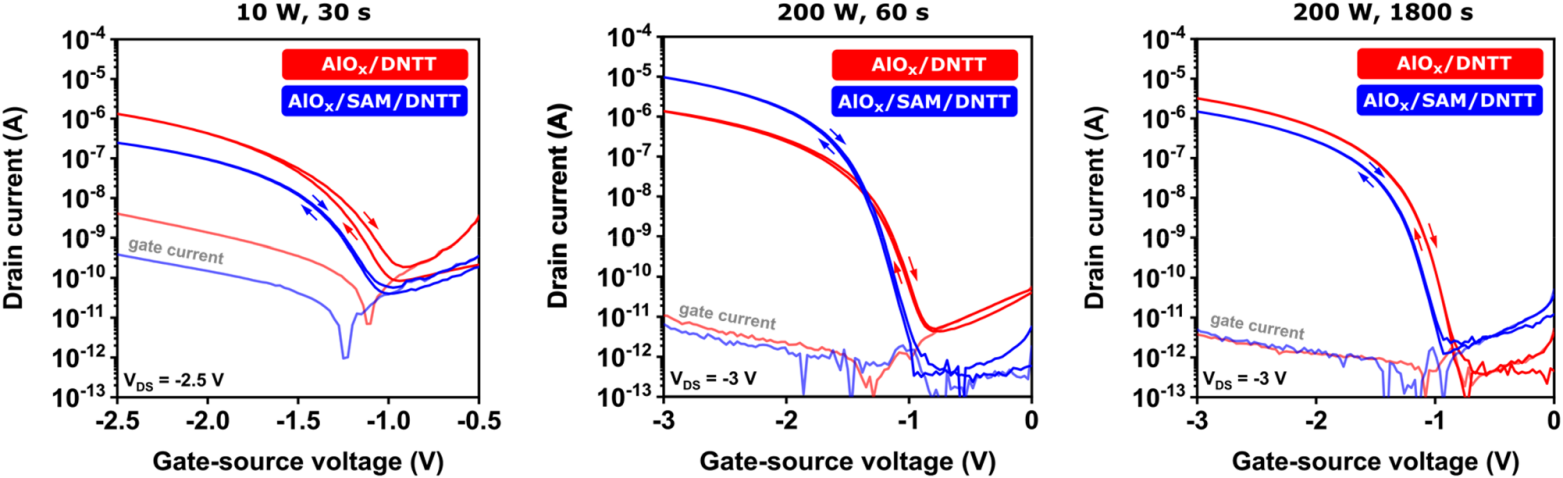
Transfer characteristics and gate currents of DNTT TFTs fabricated using either a bare-AlO_x_ gate dielectric (red curves) or a hybrid AlO_x_/SAM gate dielectric (blue curves) for three combinations of plasma power and plasma duration. (Fig. S3 shows the complete set of results.) The TFTs have a channel length of 20 µm and a channel width of 100 µm.

On the same substrates as the capacitors discussed above, we also fabricated bottom-gate, top-contact DNTT TFTs with either a bare-AlO_x_ or a hybrid AlO_x_/SAM gate dielectric produced using the same fifteen combinations of plasma power and duration as discussed above. Figure 5 shows the measured transfer characteristics and gate currents of TFTs fabricated with three of these plasma-parameter combinations; the complete set of results is shown in Figs. S3 and S4.

One observation from Figs. 5 and S3 is that a plasma power of 10 W is insufficient to suppress the gate-leakage current to an acceptable level, regardless of the plasma duration and regardless of whether or not the AlO_x_ is complemented by a SAM. The minimum plasma power required to obtain AlO_x_ films with sufficient thickness and sufficient quality to limit the gate current to 10^–11^ A over the range of gate-source voltages considered here (0 to −3 V) is 50 W. For a plasma power in the range of 100 to 200 W, both the bare-AlO_x_ and the hybrid AlO_x_/SAM gate dielectric are able to provide gate currents not exceeding 10^–11^ A and on/off current ratios of 10^6^. The optimum plasma duration for this range of plasma power is dictated by whether or not the AlO_x_ is complemented by a SAM: For the bare-AlO_x_ gate dielectric, the optimum plasma duration is ≥ 300 s, whereas for the hybrid AlO_x_/SAM gate dielectric, the optimum plasma duration is in the range of 30 to 60 s (for a plasma power ranging from 100 to 200 W).

**Figure 6:**
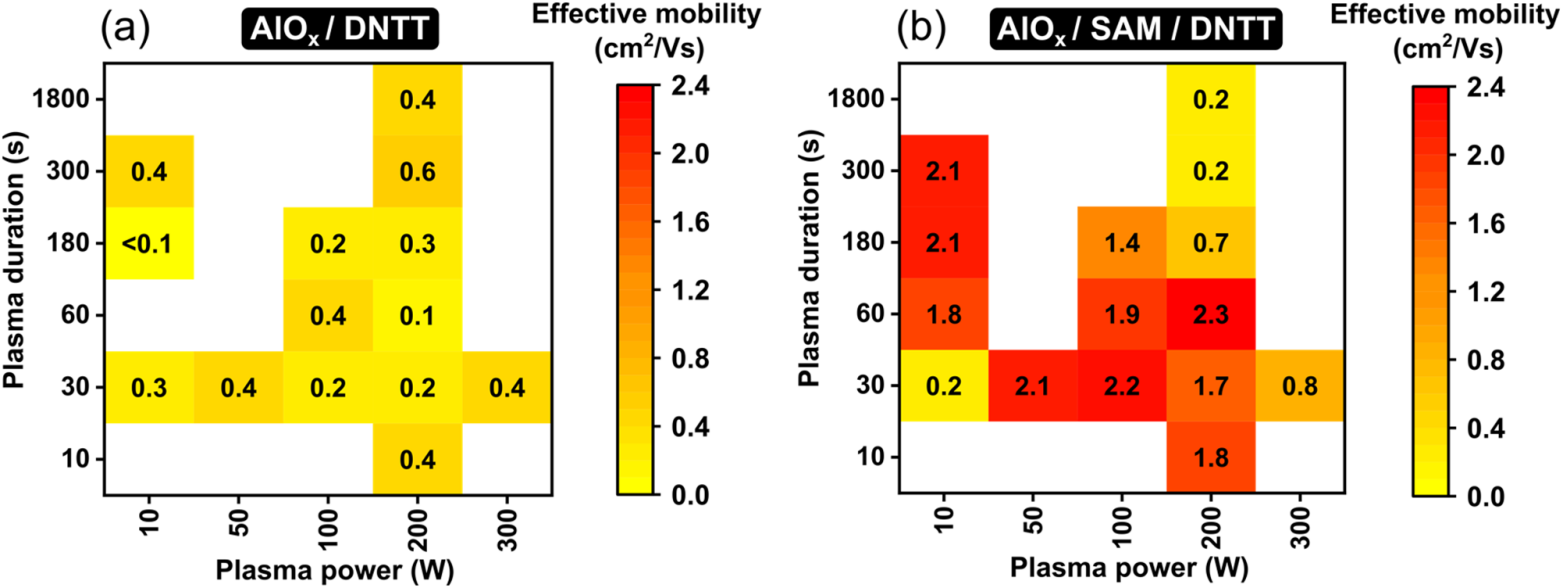
Effective charge-carrier mobilities extracted from the measured transfer characteristics of DNTT TFTs fabricated using either a bare-AlO_x_ gate dielectric (**a**) or a hybrid AlO_x_/SAM gate dielectric (**b**) as a function of plasma power and plasma duration.

In Fig. 6a, the effective charge-carrier mobility extracted from the measured transfer characteristics of the DNTT TFTs with a bare-AlO_x_ gate dielectric is plotted as a function of the plasma power and the plasma duration. As can be seen, the carrier mobility of these TFTs is rather small, between 0.1 and 0.6 cm^2^/Vs, which is possibly related to charge trapping and the formation of Fröhlich polarons, due to the fact that the organic semiconductor is in direct contact with the aluminum oxide^64,68^.

The dependence of the carrier mobility of the TFTs with a hybrid AlO_x_/SAM gate dielectric on the plasma parameters is summarized in Fig. 6b. The beneficial contribution of the SAM, in providing a hydrophobic surface with a greatly reduced density of water-related trap sites and effective screening of the organic-semiconductor film from the high-permittivity oxide, leads to notably larger carrier mobilities of up to 2.3 cm^2^/Vs. Closer inspection of Fig. 6b shows that carrier mobilities of approximately 2 cm^2^/Vs are obtained along a track from the upper left to the lower right corner of the graph, i.e., from low-power/long-duration to high-power/short-duration combinations. For parameter combinations outside of this corridor, the carrier mobilities are notably smaller, as small as 0.2 cm^2^/Vs. The reasons for this distinctive parameter-dependence pattern will be elucidated in the following section.

### Surface properties

The performance of field-effect transistors in general and of organic TFTs in particular is greatly dependent on the properties of the interface between the semiconductor and the gate dielectric^69^. In the case of bottom-gate TFTs, as considered here, this highlights the critical importance of the properties of the gate-dielectric surface. We have thus measured the surface energy and the surface roughness of the bare-AlO_x_ and the hybrid AlO_x_/SAM dielectrics for each of the fifteen combinations of plasma power and plasma duration discussed above.

The surface energies of both the bare-AlO_x_ and the hybrid AlO_x_/SAM dielectrics show only very small variations and no systematic dependence on the plasma parameters (see Fig. S7). The surface energy of the bare-AlO_x_ dielectric varies between 61 and 71 mJ/m^2^, and that of the hybrid AlO_x_/SAM dielectric between 23 and 26 mJ/m^2^, similar to previous reports^38,43^.

**Figure 7:**
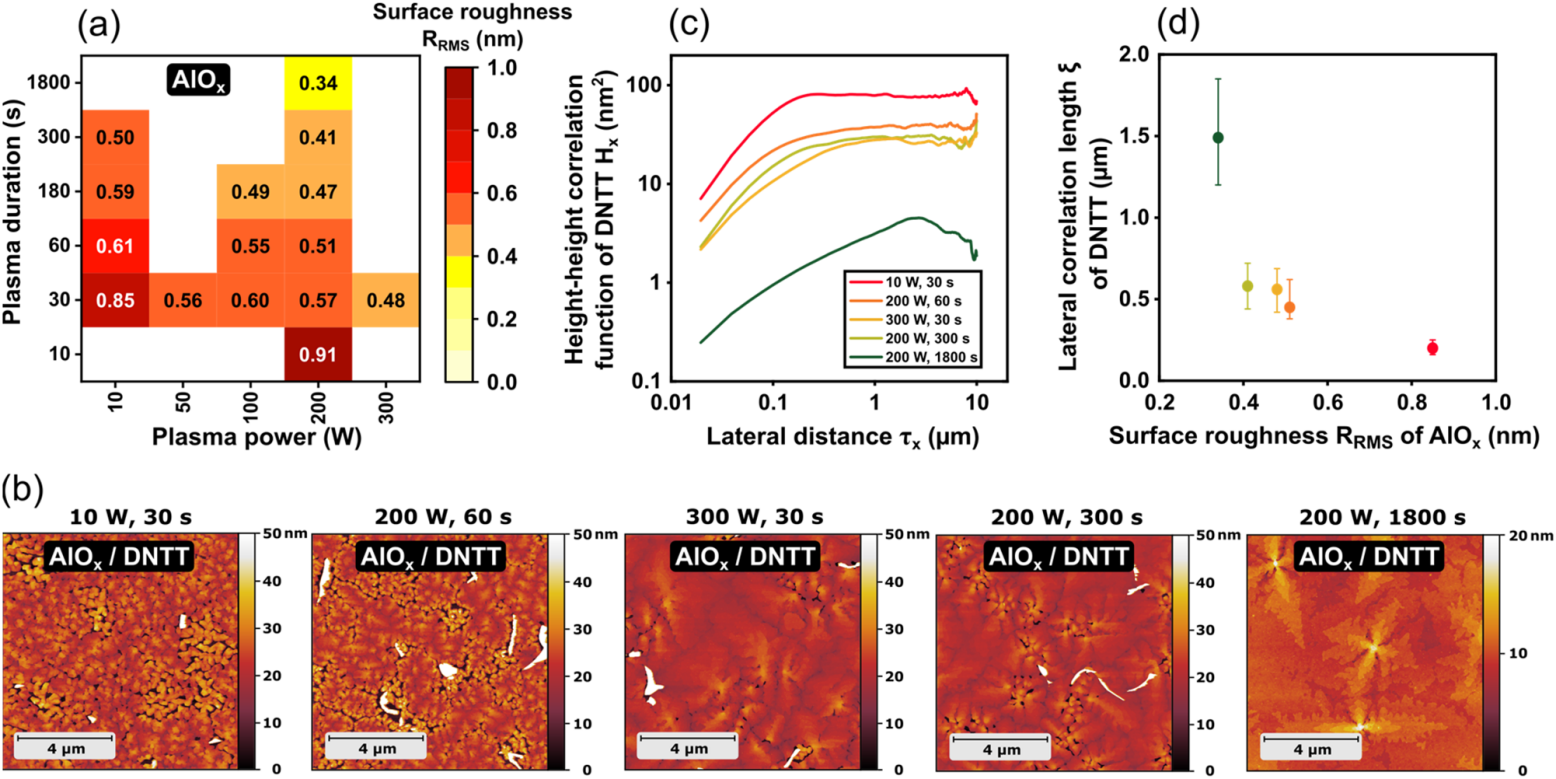
(**a**) Root-mean-square surface roughness of bare-AlO_x_ dielectrics as a function of plasma power and plasma duration. (**b**) AFM images of DNTT films deposited onto bare-AlO_x_ dielectrics for five combinations of plasma power and plasma duration. (**c**) Height-height correlation functions (HHCF) of DNTT films deposited onto bare-AlO_x_ dielectrics for the same five combinations of plasma power and plasma duration. (**d**) Lateral correlation length ξ of the DNTT films as a function of the surface roughness of the bare-AlO_x_ dielectric.

The surface roughness, on the other hand, shows a clear correlation with the plasma parameters. Prior to the plasma-oxidation process, the vacuum-deposited aluminum has a root-mean-square (RMS) surface roughness of 0.9 nm^53^. After the plasma-oxidation process, the RMS surface roughness of the bare AlO_x_ ranges from 0.34 to 0.91 nm, depending on the plasma parameters (shown in Fig. 7a). The general trend seen in Fig. 7a is that higher plasma power and longer plasma duration lead to smoother AlO_x_ films. Given the difference between the RMS surface roughness of the aluminum prior to plasma oxidation (0.9 nm) and the RMS surface roughness of the plasma-grown AlO_x_ (0.34 to 0.91 nm), it appears that the plasma-oxidation process smoothens the surface, most prominently for sufficiently high plasma powers and sufficiently long durations. This effect was not observed in our previous study^53^, in which we measured an almost identical RMS surface roughness of 0.9 nm for both the vacuum-deposited aluminum and the dielectric. However, in this previous study we did not explore the use of high plasma powers or long plasma durations, which might explain why no smoothening was observed.

The degree of the surface roughness of the dielectric affects the properties of the organic-semiconductor film deposited onto it^53^. When DNTT is deposited onto a smooth dielectric, the DNTT film exhibits a pronounced terrace-like structure, whereas deposition onto a rough dielectric results in a notably smaller terrace size. Qualitatively, this can already be seen in the AFM images in Fig. 7b, which show the morphology of DNTT deposited onto bare-AlO_x_ dielectrics produced using different plasma parameters.

To evaluate the DNTT thin-film morphology in a quantitative manner, we have applied height-height correlation functions (HHCF), calculated from the AFM data using the following equation^70^:

2 $${\mathrm{H}}_{\mathrm{x}}\left({\uptau }_{\mathrm{x}}\right)=\frac{1}{\mathrm{N}\left(\mathrm{M}-\mathrm{m}\right)}\sum_{\mathrm{l}=1}^{\mathrm{N}}\sum_{\mathrm{n}=1}^{\mathrm{M}-\mathrm{m}}{\left({\mathrm{z}}_{\mathrm{n}+\mathrm{m},\mathrm{l}}-{\mathrm{z}}_{\mathrm{n},\mathrm{l}}\right)}^{2}$$

where N and M are the number of measured rows and columns, z is the height of a measurement point, Δx is the sampling interval along the x direction, m an integer (0 ≤ m ≤ M), and τ_x_ is the lateral distance (τ_x_ = m Δx).

The HHCF has two distinct regimes, as seen in Fig. 7c: over short lateral distances, the heights are correlated and the HHCF increases linearly with distance. Over long distances, the heights are uncorrelated and the HHCF saturates at a value proportional to 2R_RMS_^2^^71^. The lateral distance at which the crossover between the two regimes occurs is the lateral correlation length ξ. In Fig. 7d, the lateral correlation length determined for each of the DNTT films deposited onto bare AlO_x_ (Fig. 7b) is plotted as a function of the RMS surface roughness of the AlO_x_. As can be seen, the lateral correlation length of the DNTT films shows a monotonic dependence on the AlO_x_ surface roughness, increasing from 0.2 nm for the largest surface roughness to 1.5 nm for the smoothest surface. However, these trends are not reflected in the measured charge-carrier mobilities of the TFTs in which these bare-AlO_x_ films serve as the gate dielectric (Fig. 6a), since the carrier mobility in these DNTT films is greatly suppressed, presumably by charge trapping and polaronic effects resulting from the close proximity of the DNTT and the aluminum oxide.

**Figure 8:**
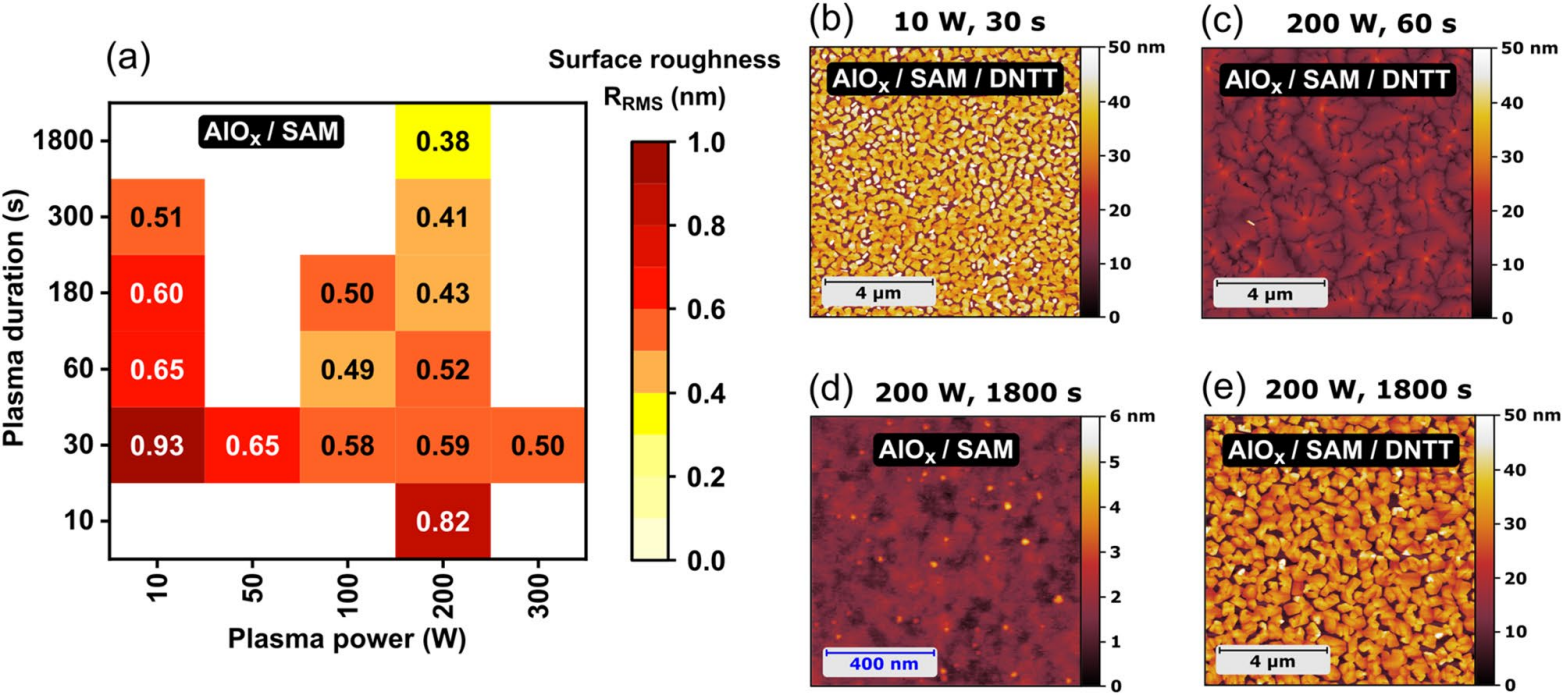
(**a**) Root-mean-square surface roughness of hybrid AlO_x_/SAM dielectrics as a function of plasma power and plasma duration. (**b**–**e**) AFM images of representative morphologies on the indicated surfaces with the indicated plasma parameters.

The RMS surface roughness of the hybrid AlO_x_/SAM dielectrics as a function of plasma power and duration is shown in Fig. 8a. Comparing Fig. 7a and Fig. 8a shows that the functionalization of the AlO_x_ surface with the SAM has no measurable effect on the roughness, i.e., the SAM covers the AlO_x_ surface in a conformal manner, as expected. The extent to which the carrier mobility of the TFTs with the hybrid AlO_x_/SAM dielectric correlates with its surface roughness can be seen by comparing Fig. 6b and Fig. 8a (see also Fig. S5 where these graphs are reproduced and the carrier mobility is plotted as a function of the RMS surface roughness): the largest RMS surface roughness (0.93 nm; obtained with low plasma power/short duration; indicated in grey in Fig. S5) leads to a disordered DNTT film with small grains (Fig. 8b) that shows a very small carrier mobility (0.2 cm^2^/Vs), as expected^53^. The largest carrier mobilities (≥ 2 cm^2^/Vs) are obtained only when the RMS surface roughness is below approximately 0.65 nm (indicated in green in Fig. S5), so that the DNTT morphology shows a pronounced terrace-like structure (Fig. 8c); this is also in line with expectations. On the other hand, the smallest RMS surface roughness (0.38 nm; obtained with medium power/long duration; indicated in blue in Fig. S5) does not lead to the largest carrier mobility, as one might have expected, but instead to a very small mobility (0.2 cm^2^/Vs). The reason for this anomaly is revealed by the AFM image of this dielectric (Fig. 8d), in which a large density of small, tall features can be seen protruding from the surface. These features do not significantly contribute to the calculated RMS surface roughness, but they influence the DNTT morphology in an unfavorable manner, as seen in Fig. 8e. The specifics of these tall features are unknown, but the fact that they appear only for long plasma durations (≥ 300 s) suggests that they mark some form of mechanical damage created on the AlO_x_ surface by prolonged plasma exposure. AFM images of DNTT films deposited onto hybrid AlO_x_/SAM dielectrics for all fifteen combinations of plasma power and plasma duration are collected in Fig. S6.

### Organic TFTs and complementary circuits on flexible plastic substrates

**Figure 9:**
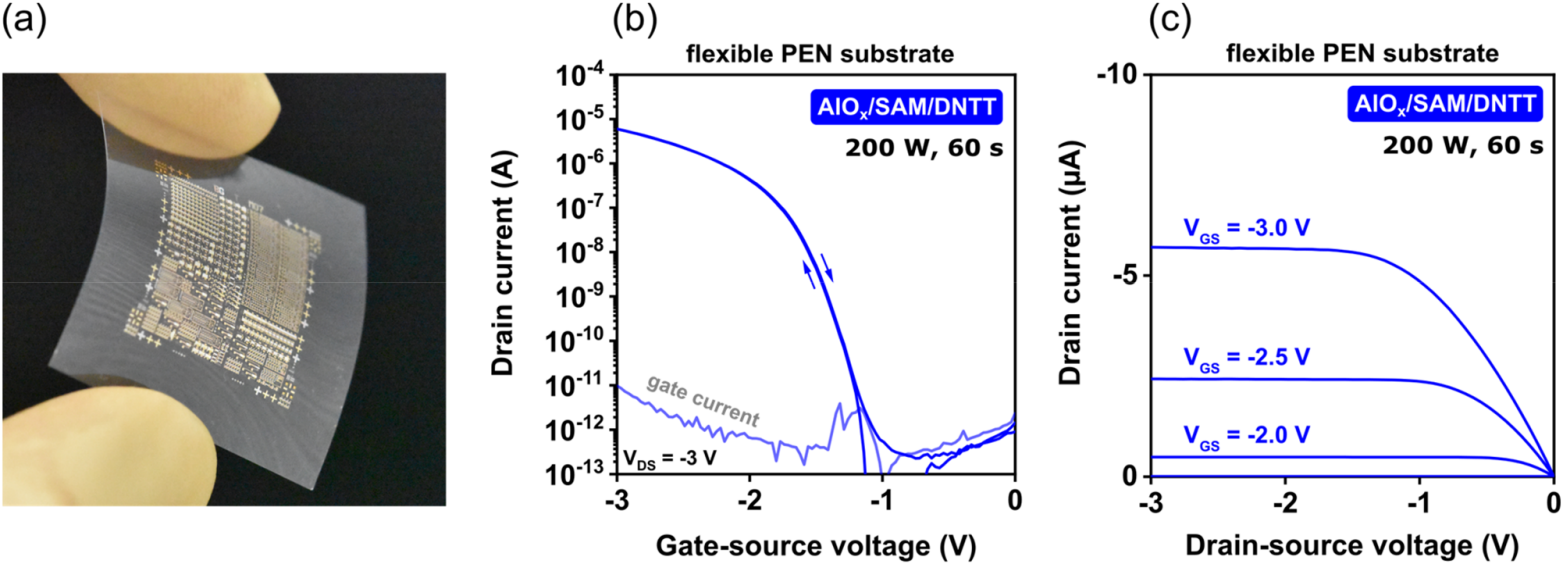
(**a**) Photograph of organic TFTs and circuits fabricated on a flexible polyethylene naphthalte (PEN) substrate. (**b**) Measured transfer and (**c**) output characteristics of a DNTT TFT with a channel length of 20 µm and a channel width of 100 µm on flexible PEN. The effective charge-carrier mobility is 2 cm^2^/Vs.

The process described above is suitable for the fabrication of organic TFTs on flexible substrates, such as plastics^15^ and paper^23^. Figure 9a shows a photograph of organic TFTs and circuits fabricated on a polyethylene naphthalate (PEN) substrate. Based on the findings reported above, a plasma power of 200 W and a plasma duration of 60 s were chosen for the preparation of the aluminum oxide as part of the gate dielectric. Figure 9b and c show the measured transfer and output characteristics of a DNTT TFT, indicating a carrier mobility of 2 cm^2^/Vs, an on/off current ratio of 10^7^ and a maximum gate current of 10^−11^ A. Results of a bias-stress measurement performed on such a TFT are summarized in Fig. S8.

**Figure 10:**
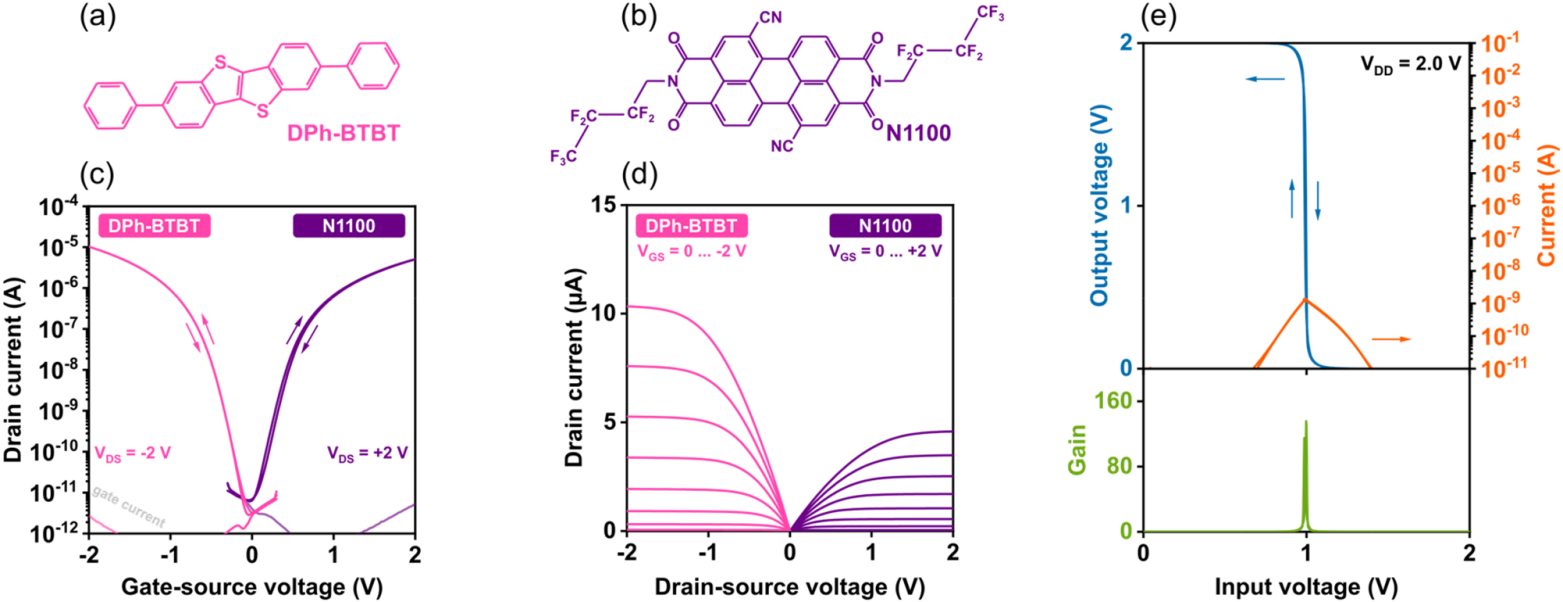
Chemical structures of the organic semiconductors (**a**) 2,7-diphenyl[1]benzothieno[3,2-b]benzothiophene (DPh-BTBT) and (**b**) (PTCDI-(CN)_2_-(CH_2_C_3_F_7_)_2_; Polyera ActivInk N1100). (**c**) Transfer and (**d**) output characteristics of a DPh-BTBT p-channel TFT and an N1100 n-channel TFT. The TFTs have a channel length of 40 µm and a channel width of 1000 µm. (**e**) Transfer characteristics of a complementary inverter based on a DPh-BTBT p-channel TFT and an N1100 n-channel TFT fabricated on a flexible PEN substrate.

Figure 10 shows the transfer and output characteristics of p-channel and n-channel organic TFTs and the transfer characteristics of a complementary inverter fabricated on flexible PEN. The AlO_x_ film was again prepared using a plasma power of 200 W and a duration of 60 s. The organic semiconductors that were used to fabricate these devices are 2,7-diphenyl[1]benzothieno[3,2-b]benzothiophene (DPh-BTBT)^15,72,73^ for the p-channel TFTs and N,N’-bis(2,2,3,3,4,4,4-heptafluorobutyl)-1,7-dicyano-perylene-(3,4:9,10)-tetracarboxylic diimide (PTCDI-(CN)_2_-(CH_2_C_3_F_7_)_2_; Polyera ActivInk N1100)^74,75^ for the n-channel TFTs. The TFTs have effective charge-carrier mobilities of 0.6 cm^2^/Vs (DPh-BTBT) and 0.2 cm^2^/Vs (N1100). At a supply voltage of 2 V, the complementary inverter has a maximum small-signal gain of 135 and a minimum noise margin of 89% of half the supply voltage, calculated according to Ref.^76^. To our knowledge, this is the largest minimum noise margin reported to date for an organic complementary inverter fabricated on a flexible substrate (see Table 1).

**Table 1:** Summary of organic complementary inverters with a small-signal gain greater than 100 and a minimum noise margin of at least 80% of half the supply voltage (V_DD_).

Reference	Substrate material	Gate-dielectric material	Gate-dielectric capacitance (nF/cm^2^)	Small-signal gain	Supply voltage (V)	Voltage-normalized gain (V^−1^)	Noise margin (of V_DD_/2) (%)	Measurement ambient
^76^	Glass	AlO_x_/SAM	700	435	2	218	80	Air
^78^	Glass	Anodic Ta_2_O_5_	140	500	5	100	84	Vacuum
^79^	Glass	AlO_x_/SAM	700	120	3	40	80	Air
^80^	Glass	AlO_x_/SAM	700	376	3	125	80	Air
^81^	Glass	Al_2_O_3_/TMSC	65	500	4	125	92.5	Glove box
^81^	Glass	Al_2_O_3_/TMSC	65	740	5	148	85	Glove box
^15^	Flexible PEN	AlO_x_/SAM	700	180	1	180	84	Air
^82^	Flexible PEN	Al_2_O_3_/PS	26.5	170	5	34	80	Glove box
^83^	Flexible PEN	CVD polymer	70	118	3	39	80	Glove box
This work	Flexible PEN	AlO_x_/SAM	700	135	2	68	89	Air

## Conclusion

Ultrathin, high-capacitance hybrid gate dielectrics based on oxygen-plasma-grown aluminum oxide films in combination with an alkylphosphonic acid SAM are useful for the realization of low-voltage organic TFTs. Depending on the plasma power and the duration of the plasma exposure, the thickness of the plasma-grown AlO_x_ films can be tuned to values between approximately 4.3 and 7.3 nm. The lower limit of the range of accessible thicknesses is dictated by the thickness of the native aluminum oxide (approximately 3 nm) that forms spontaneously when the substrates are exposed to ambient air prior to the plasma-oxidation process, while the upper boundary is set by the inherently self-limiting oxidation kinetics of aluminum oxide. The relative permittivity of the oxygen-plasma-grown AlO_x_ films is approximately 8 ± 0.2, and the capacitance of the bare-AlO_x_ films thus falls into the range from 1 to 1.6 µF/cm^2^. By allowing a high-quality monolayer of an alkylphosphonic acid with medium alkyl-chain length to self-assemble on the freshly grown AlO_x_ surface, a hybrid AlO_x_/SAM dielectric is obtained, the capacitance of which varies between 0.7 and 0.8 µF/cm^2^, depending on the plasma parameters. The leakage-current density through the hybrid AlO_x_/SAM dielectrics was found to be smaller by about an order of magnitude than the current density through bare-AlO_x_ dielectrics. For example, for a plasma power of 200 W and a plasma duration of 60 s, the leakage-current density through the hybrid AlO_x_/SAM dielectric drops below 10^–6^ A/cm^2^ at voltages of ± 3 V, confirming the beneficial effect of the SAM in improving the insulating properties of the gate dielectric for low-power organic TFTs.

The effective charge-carrier mobility of DNTT TFTs with a bare-AlO_x_ gate dielectric is no greater than 0.6 cm^2^/Vs, and shows little dependence on the plasma parameters. In TFTs with a hybrid AlO_x_/SAM dielectric, carrier mobilities ranging from 1.8 to 2.3 cm^2^/Vs were obtained for a number of favorable combinations of plasma power and plasma duration that produce AlO_x_ films with small surface roughness and thus promote the formation of high-quality SAMs and well-ordered DNTT films on these dielectrics. An important finding is that while the properties of the plasma-grown AlO_x_ films can be tuned over a certain range without negatively affecting the charge-transport properties in the organic-semiconductor films deposited onto them, this is only true as long as the plasma power and the plasma duration do not exceed values beyond which the quality of the plasma-grown oxide films suffers from surface damage. The largest carrier mobility equaling 2.3 cm^2^/Vs was obtained for a plasma-parameter combination of 200 W and 60 s.

This work highlights the properties of oxygen-plasma-grown aluminum oxide films as part of high-capacitance gate dielectrics in low-voltage organic transistors and identifies the optimum process parameters for their fabrication.

## Experimental section

### Fabrication of capacitors and TFTs on silicon substrates

Metal–insulator–metal capacitors and inverted staggered (bottom-gate, top-contact) TFTs were fabricated on silicon substrates coated with 100-µm-thick thermally grown silicon dioxide. For the bottom electrode of the capacitors and the gate electrode of the TFTs, aluminum with a thickness of 30 nm and a root-mean-square surface roughness of less than 1 nm (measured by AFM^53^) was deposited by thermal evaporation in vacuum with a rate of about 20 Å/s. AlO_x_ films were produced by plasma oxidation in an Oxford Instruments ProLab100 Cobra system in pure oxygen with a partial pressure of 0.01 mbar using the capacitively coupled plasma mode with an excitation frequency of 13.56 MHz. We fabricated substrates with fifteen different combinations of plasma power (ranging from 10 to 300 W) and duration (ranging from 10 to 1800 s). After the plasma oxidation, each substrate was cleaved into two halves. One half was immersed into a 2-propanol solution of *n*-tetradecylphosphonic acid (PCI Synthesis, Newburyport, MA, USA) to form a self-assembled monolayer and hence a hybrid AlO_x_/SAM dielectric. The other half remained without SAM (bare-AlO_x_ dielectric). Onto both halves of each substrate, the small-molecule semiconductor dinaphtho[2,3-b:2′,3′-f]thieno[3,2-b]thiophene (DNTT; Sigma Aldrich) was deposited by thermal sublimation in vacuum with a deposition rate of 0.3 Å/s and with a nominal thickness of 25 nm. During the DNTT deposition, the substrate was held at a constant temperature of 80 °C. For the top electrode of the capacitors and the source/drain contacts of the TFTs, gold with a thickness of 30 nm was deposited by thermal evaporation in vacuum with a rate of 0.3 Å/s. The metals and the DNTT were patterned using polyimide shadow masks (CADiLAC Laser, Hilpoltstein, Germany). The capacitors have an area of 200 µm × 200 µm. The TFTs have a channel length of 20 µm and a channel width of 100 µm. For each of the fifteen combinations of plasma power and plasma duration, capacitors and TFTs were fabricated on the same substrate to minimize the effects of unintentional parameter variations.

### Fabrication of TFTs and complementary inverters on flexible substrates

Polyethylene naphthalate (PEN) with a thickness of 125 µm (Teonex Q65 PEN; kindly provided by William A. MacDonald, DuPont Teijin Films, Wilton, U.K.) was used as a flexible substrate. Aluminum gate electrodes (thickness: 30 nm) and AlO_x_ films (plasma power: 200 W, plasma duration: 60 s) were prepared as on the silicon substrates. For DNTT TFTs, an *n*-tetradecylphosphonic acid SAM was prepared as on the silicon substrates. For TFTs based on the semiconductors 2,7-diphenyl[1]benzothieno[3,2-b]benzothiophene (DPh-BTBT; Sigma Aldrich) and N,N’-bis(2,2,3,3,4,4,4-heptafluorobutyl)-1,7-dicyano-perylene-(3,4:9,10)-tetracarboxylic diimide (PTCDI-(CN)_2_-(CH_2_C_3_F_7_)_2_; ActivInk N1100; Polyera Corp., Skokie, IL, U.S.A.), a mixed SAM composed of 25% *n*-octadecylphosphonic acid and 75% 12,12,13,13,14,14,15,15,16,16,17,17,18,18,18-pentadecafluoroctadecylphosphonic acid (synthesized by Matthias Schlörholz, Heidelberg, Germany) was prepared^15^. Source and drain contacts were prepared as on the silicon substrates. The DNTT TFTs have a channel length of 20 µm and a channel width of 100 µm. The DPh-BTBT and N1100 TFTs have a channel length of 40 µm and a channel width of 1000 µm.

### TEM characterization

The TEM specimen was prepared on a silicon substrate by repeating the deposition of 30-nm-thick aluminum and the plasma-induced oxidation of its surface five times, each time with a different combination of plasma power and plasma duration. Preparing all five AlO_x_ films on the same substrate, rather than on five separate substrates, was helpful in minimizing the time required for thinning the specimen in preparation for cross-sectional microscopy. The TEM specimen was fabricated by conventional focused ion beam (FIB) lift-out using a Thermo Fisher Scientific FEI Scios DualBeam instrument equipped with a gallium source. A platinum strip was deposited to protect the films from ion-beam damage. A lamella with a size of approximately 20 µm × 10 µm was released from the substrate and glued to a copper TEM lift-out grid. The lamella was then thinned to a thickness of less than 100 nm using an acceleration voltage of 30 kV and an ion current of initially 500 pA that was successively decreased to 100 pA. Afterwards, the lamella was cleaned using a low-voltage cleaning step with an acceleration voltage of 5 kV and an ion current of 48 pA to remove gallium-beam damage. The TEM image was recorded in bright-field (BF) mode using a Philips CM-200 FEG TEM operated with an acceleration voltage of 200 kV.

### Electrical characterization

All electrical measurements were performed in ambient air at room temperature under yellow laboratory light. The capacitance measurements were performed using a Hameg HM8118 LCR meter by applying an alternating voltage with an amplitude of 0.2 V and a frequency of 1 kHz. The current–voltage measurements were performed using an Agilent 4156C Semiconductor Parameter Analyzer and the measurement software “SweepMe!” (https://sweep-me.net). The effective charge-carrier mobility was calculated by fitting the following equation to the measured transfer curve:

3 $${\upmu }_{\mathrm{eff}}= \frac{2\mathrm{ L}}{{\mathrm{C}}_{\mathrm{diel}}\mathrm{ W}} {\left(\frac{\partial \sqrt{{\mathrm{I}}_{\mathrm{D}}}}{{\partial \mathrm{V}}_{\mathrm{GS}}}\right)}^{2}$$

where I_D_ is the drain current, V_GS_ the gate-source voltage, C_diel_ the unit-area capacitance of the gate dielectric, L the channel length and W the channel width.

### Surface characterization

AFM images were recorded in air using a Bruker Dimension Icon Atomic Force Microscope in peak force tapping mode (for the DNTT films) or in tapping mode (for the dielectrics). Data processing was performed using the AFM analysis software Gwyddion. Static contact-angle measurements were performed using a Krüss contact angle measurement system. The contact angles of water and hexadecane on the AlO_x_ dielectrics were measured immediately after the plasma treatment of the aluminum films. The contact angles of water and hexadecane on the AlO_x_/SAM hybrid dielectrics were measured immediately after the SAM treatment. The surface energies were calculated using the Owens–Wendt method^77^.

## Supplementary Information

Supplementary Information
