# Supplementary material for: Optimizing the plasma oxidation of aluminum gate electrodes for ultrathin gate oxides in organic transistors
Source: Sci Rep. 2021 Mar 18;11:6382. doi: 10.1038/s41598-021-85517-7 (PMC7973517; doi:10.1038/s41598-021-85517-7)
Supplement: Supplementary file 1 — Supplementary Information [file 41598_2021_85517_MOESM1_ESM.pdf]

Supplementary Information

Optimizing the plasma oxidation of aluminum gate electrodes for ultrathin gate oxides in organic transistors

Michael Geiger, Marion Hagel, Thomas Reindl, Jürgen Weis,
R. Thomas Weitz, Helena Solodenko,
Guido Schmitz, Ute Zschieschang, Hagen Klauk, Rachana Acharya

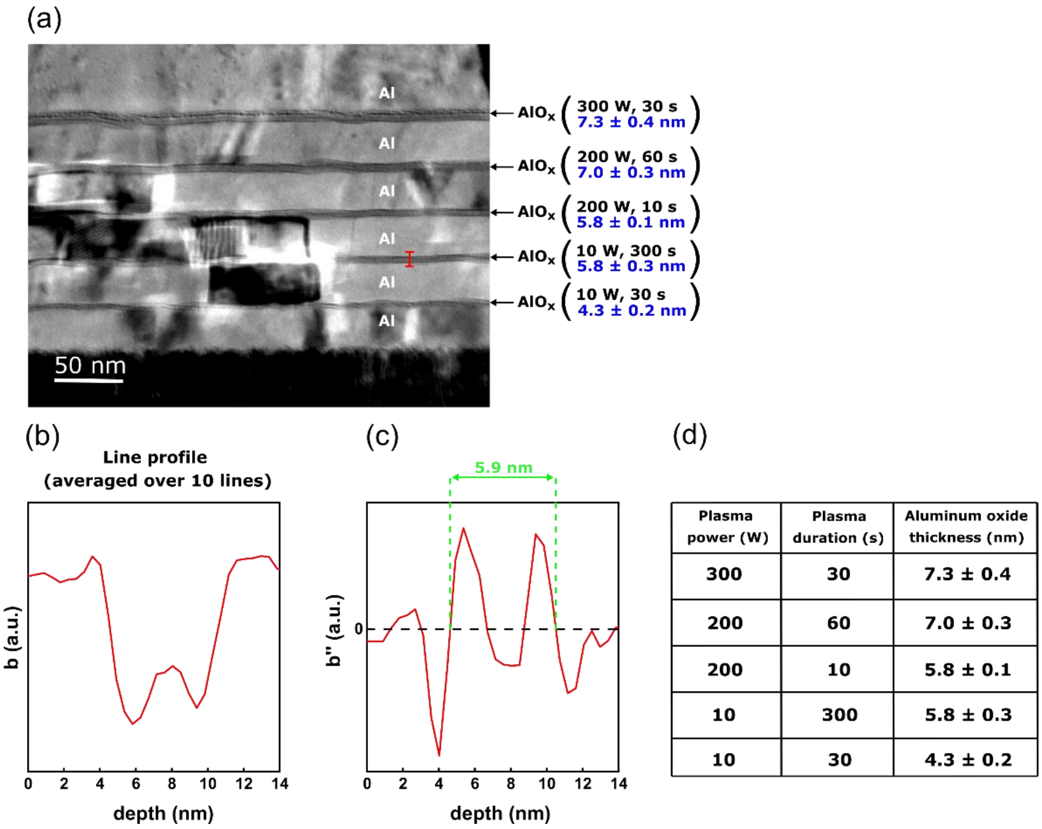

(d)

Plasma power (W)	Plasma duration (s)	Aluminum oxide thickness (nm)
300	30	7.3 ± 0.4
200	60	7.0 ± 0.3
200	10	5.8 ± 0.1
10	300	5.8 ± 0.3
10	30	4.3 ± 0.2

Figure S1: Analysis of the cross-sectional TEM image to extract the thicknesses of the AlO_x films produced using five different combinations of plasma power and plasma duration. From the digital data representing the TEM image (a), a depth profile (having a line width of 10 pixels) through the AlO_x film (starting and ending a few nanometers above and below the Al/AlO_x interfaces) representing the average pixel brightness (denoted as "b") was extracted using the image software Gwyddion (b). In the depth profile, the materials Al and AlO_x are distinguished by larger and smaller values of the pixel brightness b, respectively. The second derivative of b with respect to the depth was calculated and plotted as a function of depth to identify the points of inflection ($b'' = 0$) in the depth profile, and the distance between these points was measured as the thickness of the AlO_x film (c). For each of the five AlO_x films, this process was repeated 10 times in various locations across the TEM image, and the averaged results were tabulated (d).

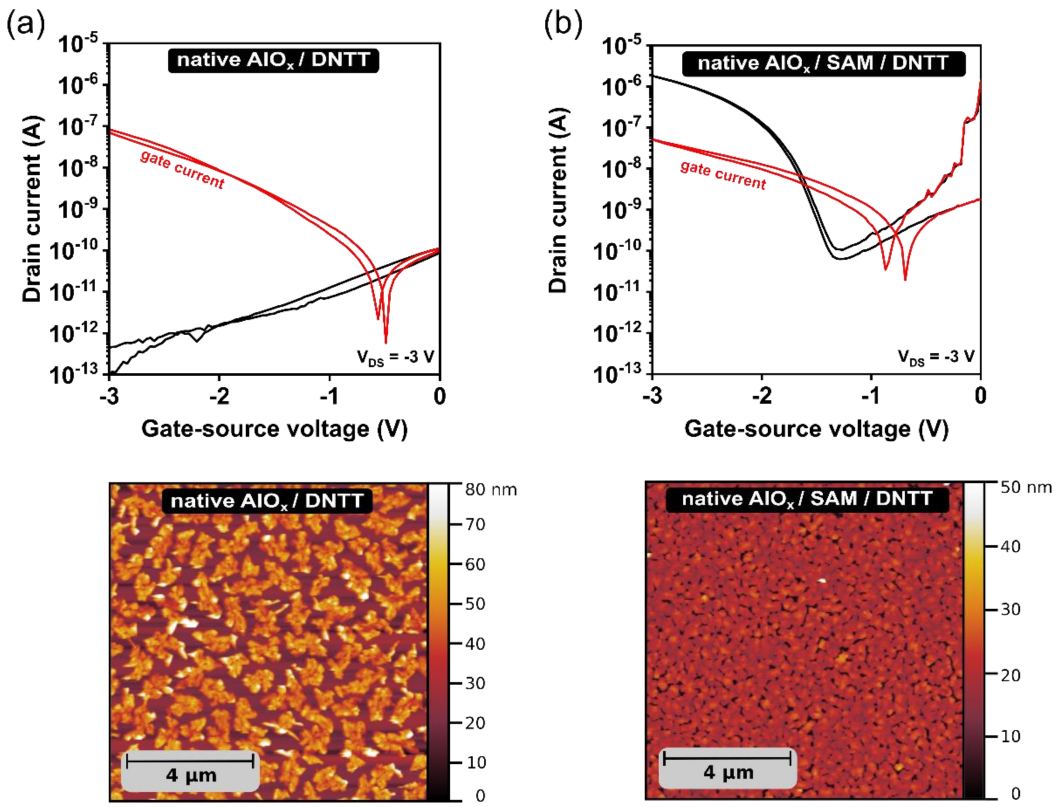

Figure S2: Transfer characteristics and gate currents of DNTT TFTs fabricated using native aluminum oxide (obtained without plasma process) either as the gate dielectric (a) or as part of a hybrid AlO_x/SAM gate dielectric (b). As can be seen, the native aluminum oxide alone is insufficient as a gate dielectric, as the TFTs either do not show a field effect (a) or suffer from gate currents exceeding 10 nA at a gate-source voltage of -2 V. AFM images of DNTT films deposited onto the bare native AlO_x and the hybrid AlO_x/SAM gate dielectric are also shown.

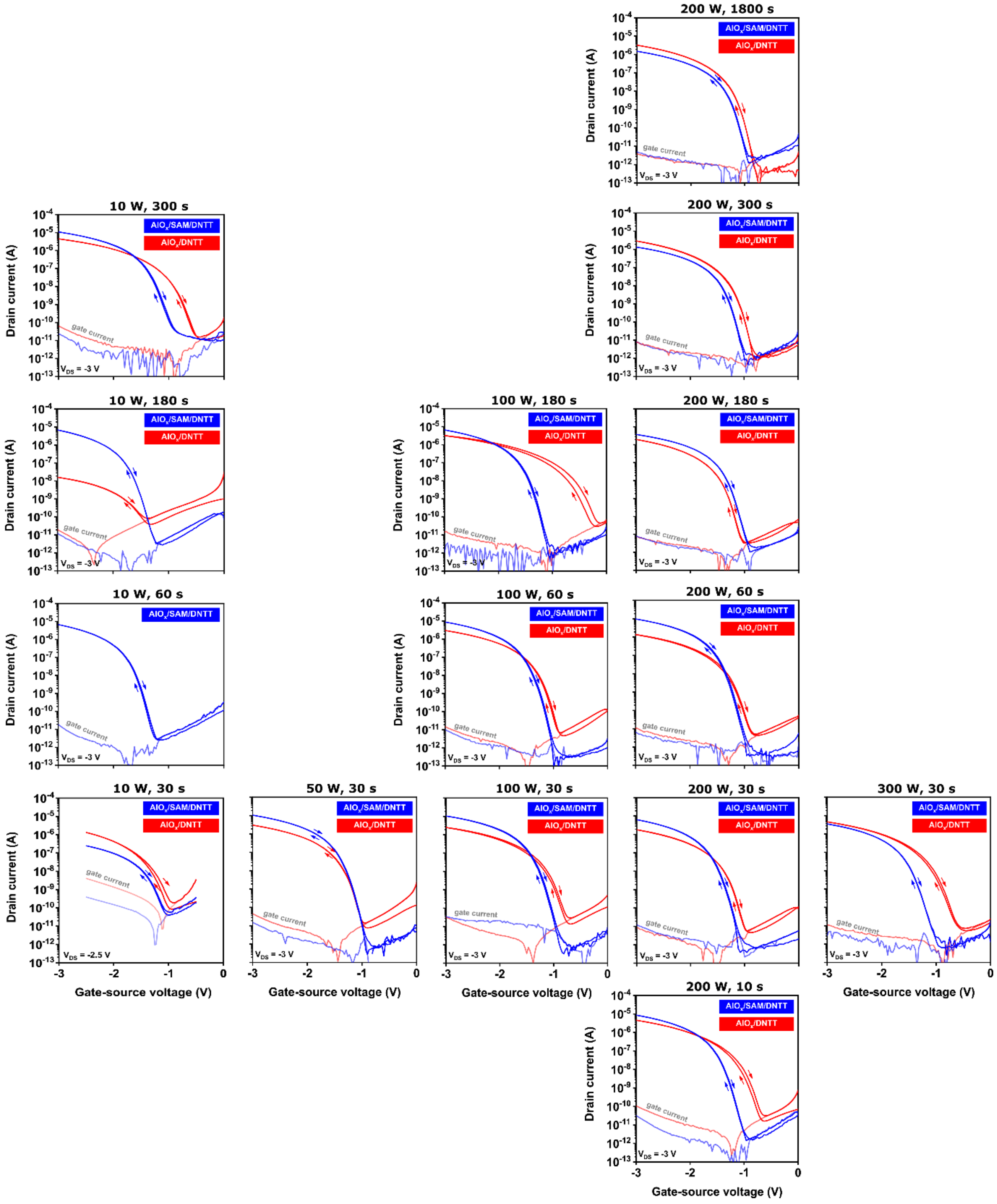

Figure S3: Transfer characteristics and gate currents of DNTT TFTs fabricated using either a bare-AlO_x gate dielectric (red curves) or a hybrid AlO_x/SAM gate dielectric (blue curves) for all fifteen combinations of the plasma power and plasma duration.

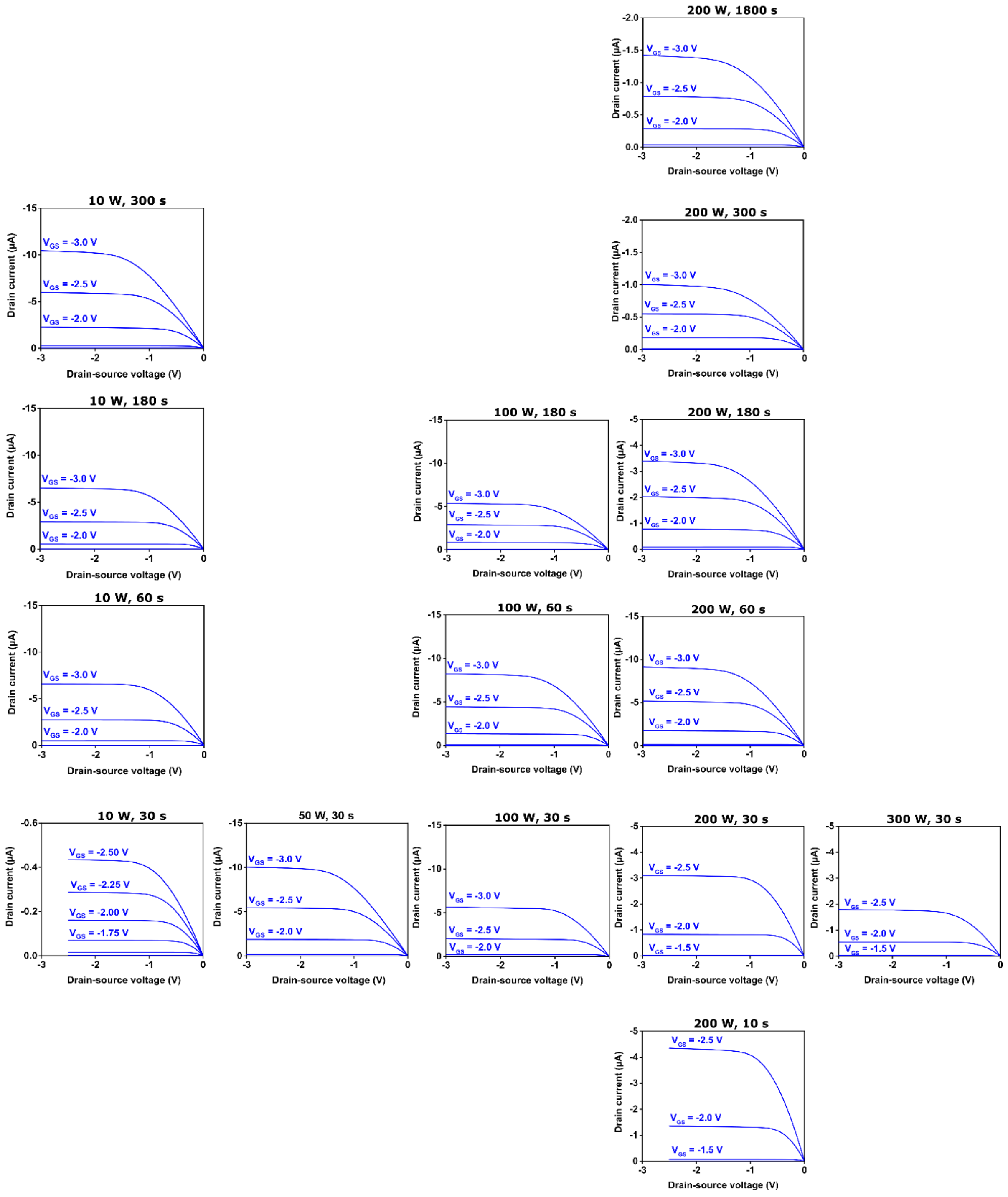

Figure S4: Output characteristics of DNTT TFTs fabricated using a hybrid AlO_x/SAM gate dielectric for all fifteen combinations of plasma power and plasma duration.

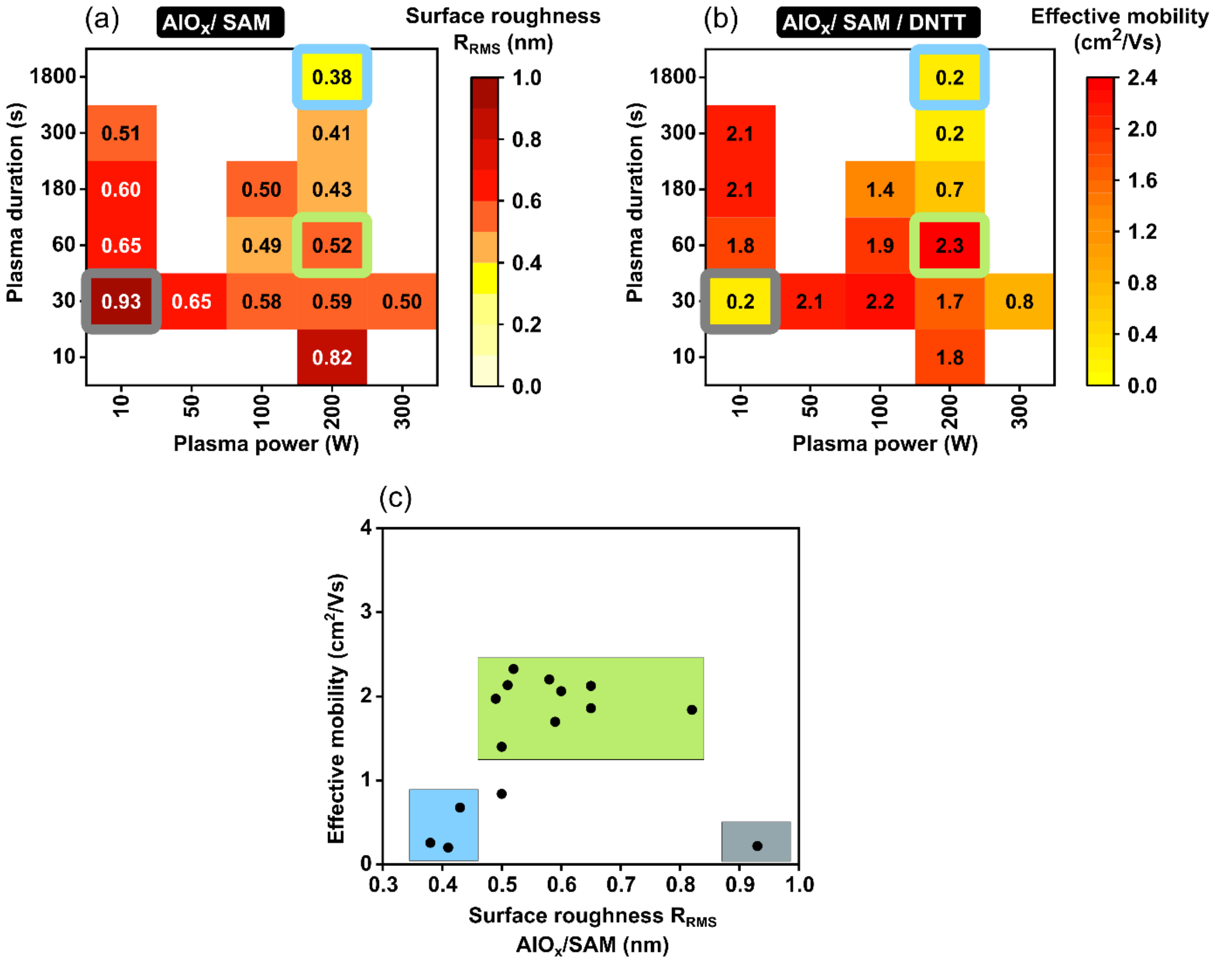

Figure S5: (a) Root-mean-square surface roughness of hybrid AlO_x/SAM dielectrics as a function of plasma power and plasma duration. (b) Carrier mobilities extracted from the measured transfer characteristics of DNTT TFTs fabricated using a hybrid AlO_x/SAM gate dielectric as a function of plasma power and plasma duration. (c) Carrier mobility plotted as a function of the RMS surface roughness.

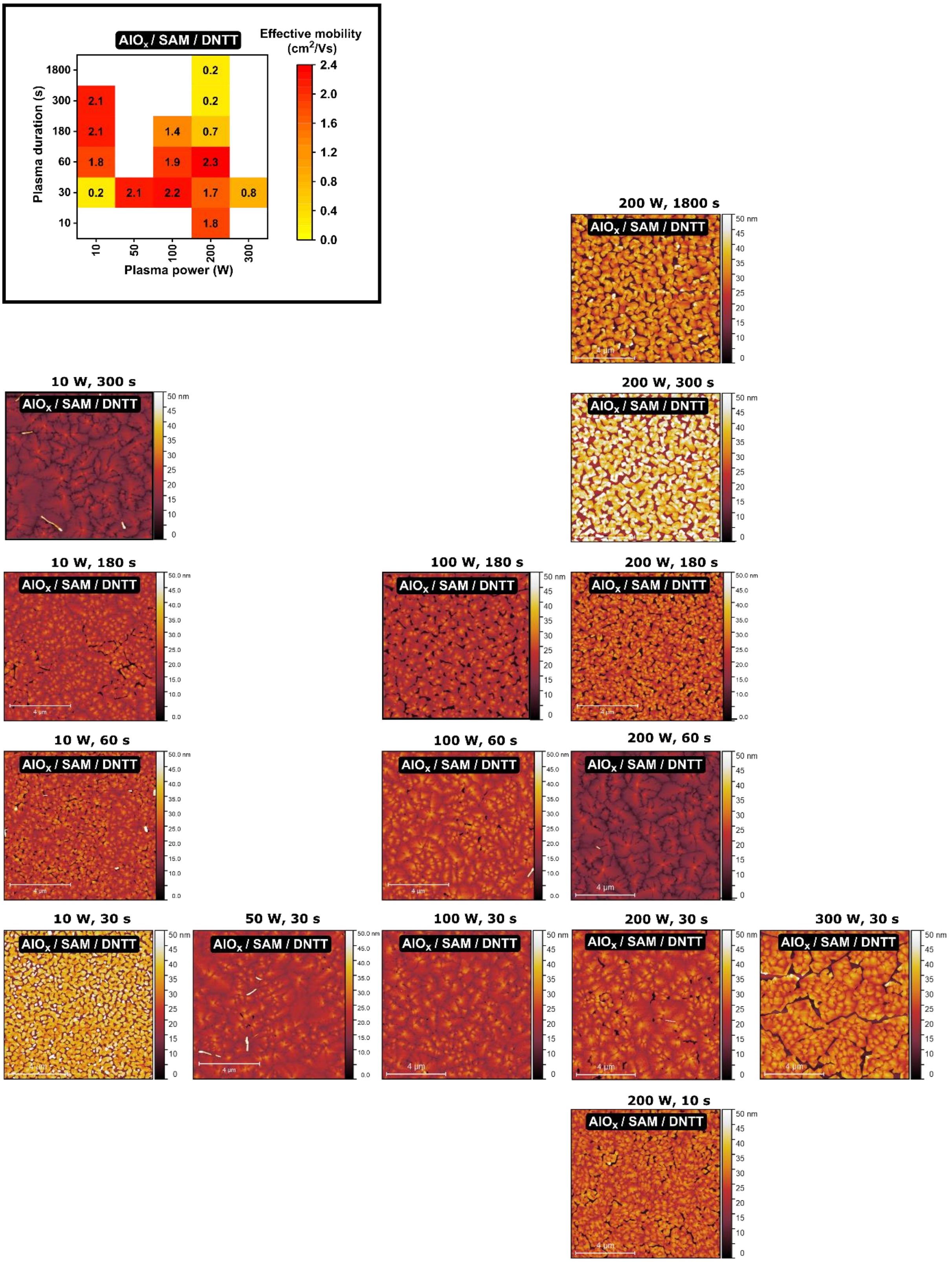

Figure S6: AFM images of DNTT films deposited onto hybrid AlO_x/SAM dielectrics for all fifteen combinations of plasma power and plasma duration.

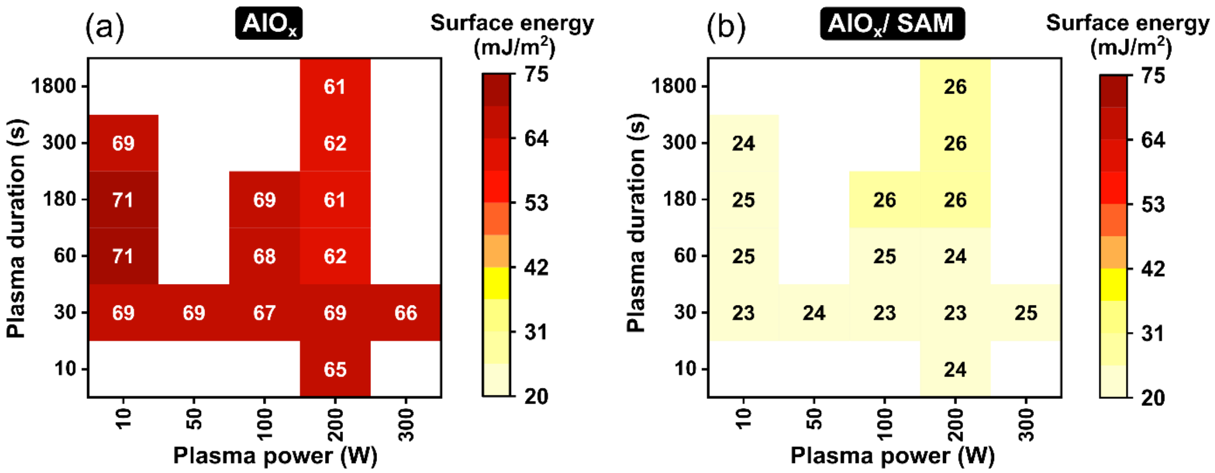

Figure S7: Surface energy of bare-AlO_x dielectrics (a) and hybrid AlO_x/SAM dielectrics as a function of plasma power and plasma duration.

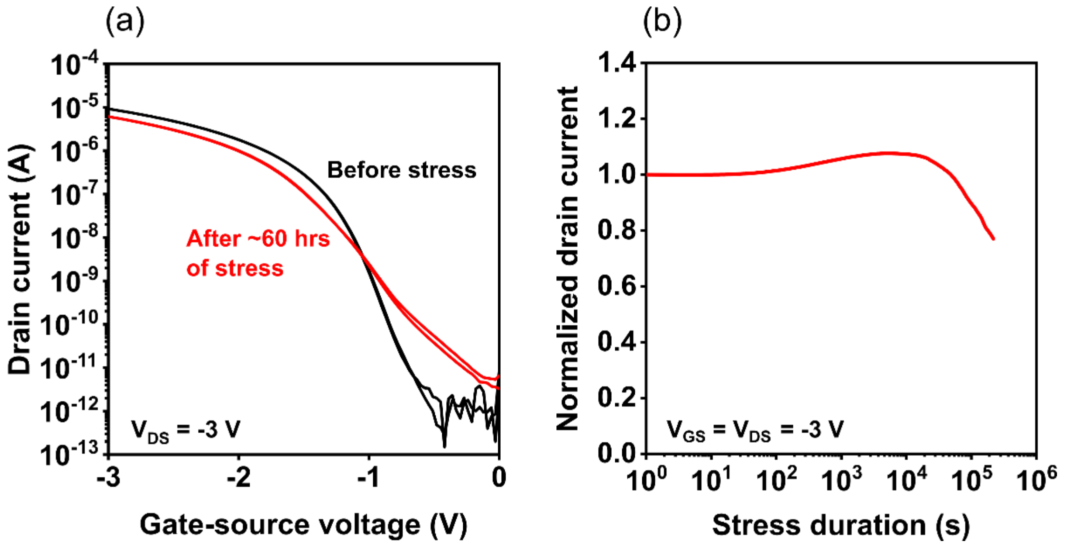

Figure S8: (a) Transfer characteristics of a DNTT TFT measured before and after bias stress. (b) Drain current measured continuously over a period of 60 hours during bias stress with gate-source and drain-source voltages of -3 V.
